# Metastatic Lung Adenocarcinoma Diagnosed by Thyroid Biopsy: A Case Report

**DOI:** 10.7759/cureus.67693

**Published:** 2024-08-24

**Authors:** Nishat A Momin, Hannah G Luk, Jing He, Cecilia Clement, Sepehr Shabani

**Affiliations:** 1 Otolaryngology - Head and Neck Surgery, University of Texas Medical Branch, Galveston, USA; 2 John Sealy School of Medicine, University of Texas Medical Branch, Galveston, USA; 3 Pathology, University of Texas Medical Branch, Galveston, USA

**Keywords:** thyroid transcription factor 1, thyroid, secondary neoplasm, lung adenocarcinoma, metastasis

## Abstract

Lung cancer metastasis to the thyroid gland is a rare occurrence. We report a rare presentation of metastatic lung adenocarcinoma diagnosed by thyroid biopsy during a tracheostomy in a 35-year-old female. A 35-year-old female with a history of epilepsy, hypothyroidism, and 15-pack-year smoking presented with four months of increasing neck swelling. The patient reported no airway symptoms upon admission. Initial flexible laryngoscopy revealed supraglottic edema. Workup including CT neck and chest revealed diffuse bilateral cervical lymphadenopathy, diffusely enlarged thyroid gland without any nodules or masses, and mediastinal lymphadenopathy with no obvious lung masses or nodules. Excisional right axillary nodal biopsy as well as right supraclavicular biopsy showed metastatic carcinoma with an equivocal staining pattern favoring lung adenocarcinoma versus thyroid carcinoma. During inpatient admission, the patient began having increasing dyspnea with flexible laryngoscopy revealing worsening supraglottic mucosal edema. The patient subsequently underwent tracheostomy with excisional thyroid biopsy due to concern for malignancy. Intraoperatively, the strap muscles were found to be firmly adhered to the underlying thyroid gland. Dissection of the thyroid isthmus revealed thickened tissue. The final pathology of the thyroid biopsy revealed metastatic adenocarcinoma, consistent with lung primary. It is important to keep in mind that, although rare, the thyroid gland may be a site of metastasis for primary lung adenocarcinoma. Prompt recognition and understanding of this possible event are key to achieving adequate disease control.

## Introduction

Metastasis to the thyroid gland is an uncommon occurrence with a generally poor prognosis [1]. It has a rare incidence of 0.1% to 3% in clinical series [2]. Within these clinical studies, the most common primary tumor to metastasize is renal cell carcinoma, followed by lung carcinoma [2]. A larger proportion of metastasis to the thyroid gland is observed in autopsy series with incidental findings ranging from 4.4% to 24% [2]. Within these autopsy studies, primary tumors are predominated by lung, breast, and colon carcinomas [2]. Of the lung carcinomas that metastasize to the thyroid gland, the most common histological subtypes are adenocarcinoma and squamous cell carcinoma [3]. These features of the primary tumor appear to be closely related to the overall prognosis of metastasis to the thyroid [4,5]. 

Metastasis to the thyroid is more commonly seen in the fifth to seventh decade of life, with a median age between 54 and 68 years [4]. No obvious gender predominance has been noted [3]. Diagnosis can be challenging due to the variety of potential primary tumor origins and histologic types [2]. Additionally, clinical history can lack indications of a primary prior neoplasm and the interval time between diagnosis of the primary tumor and detection of metastasis to the thyroid can be prolonged [2]. 

Secondary neoplasms of the thyroid gland can present as solitary or diffuse and may be variably symptomatic or detectable [2]. Only a small proportion of cases present clinically with a palpable mass [4]. More commonly, 28%-54% of cases are discovered incidentally in asymptomatic patients [3]. Symptomatic patients with obstructive mass effects, lymphadenopathy and thyroid dysfunction are less common [3].

An effective tool for detection and diagnosis is fine needle aspiration biopsy (FNAB) [2]. FNAB cytology reveals high cellularity, focal tumor necrosis, and high-grade nuclear features [1]. Of malignant thyroid samples, 1.9% of FNABs are diagnosed as secondary thyroid neoplasms [3]. 

This case report describes a unique and rare presentation of metastatic lung adenocarcinoma diagnosed by thyroid biopsy of a 35-year-old female, including a description of symptom onset, and subsequent workup.

## Case presentation

The patient is a 35-year-old female with hypothyroidism, epilepsy, and 15-pack-year smoking history who presented with progressively worsening bilateral neck swelling, dysphagia, and pressure sensation in the neck for four months. Physical exam revealed bilateral diffuse cervical lymphadenopathy including levels I-IV, most notable at right level II. Flexible laryngoscopy at presentation revealed moderate supraglottic edema. 

Of note, two months prior to her initial presentation, the patient had a right axillary lymph node excisional biopsy performed. Pathology was consistent with metastatic carcinoma. Per the outside report, immunostains of tumor cells reported diffusely positive for thyroid transcription factor 1 (TTF-1), high-molecular-weight cytokeratin, and low-molecular-weight cytokeratin. 

**Figure 1:**
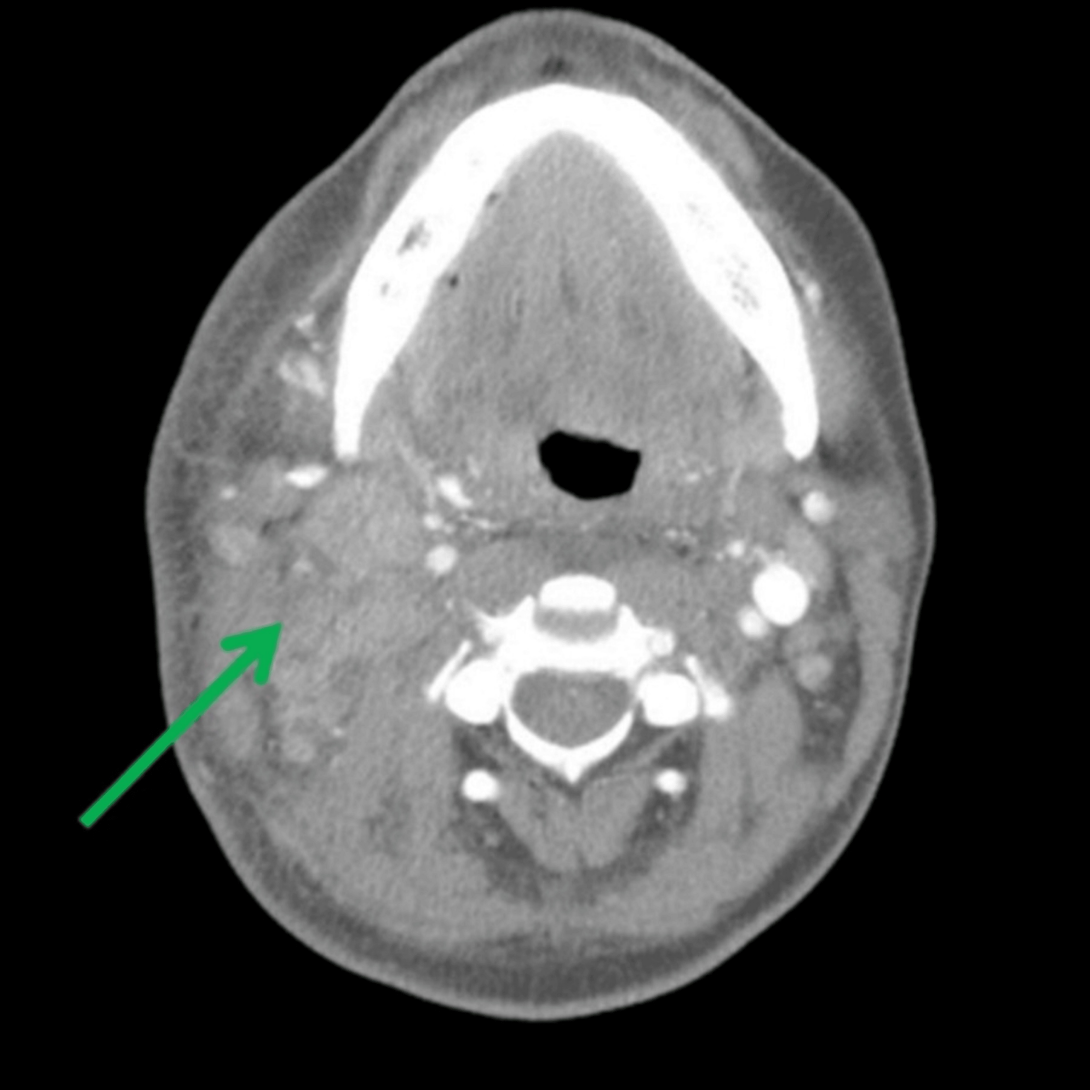
CT neck with contrast demonstrating increased diffuse metastatic cervical lymphadenopathy right (green arrow) greater than left.

**Figure 2:**
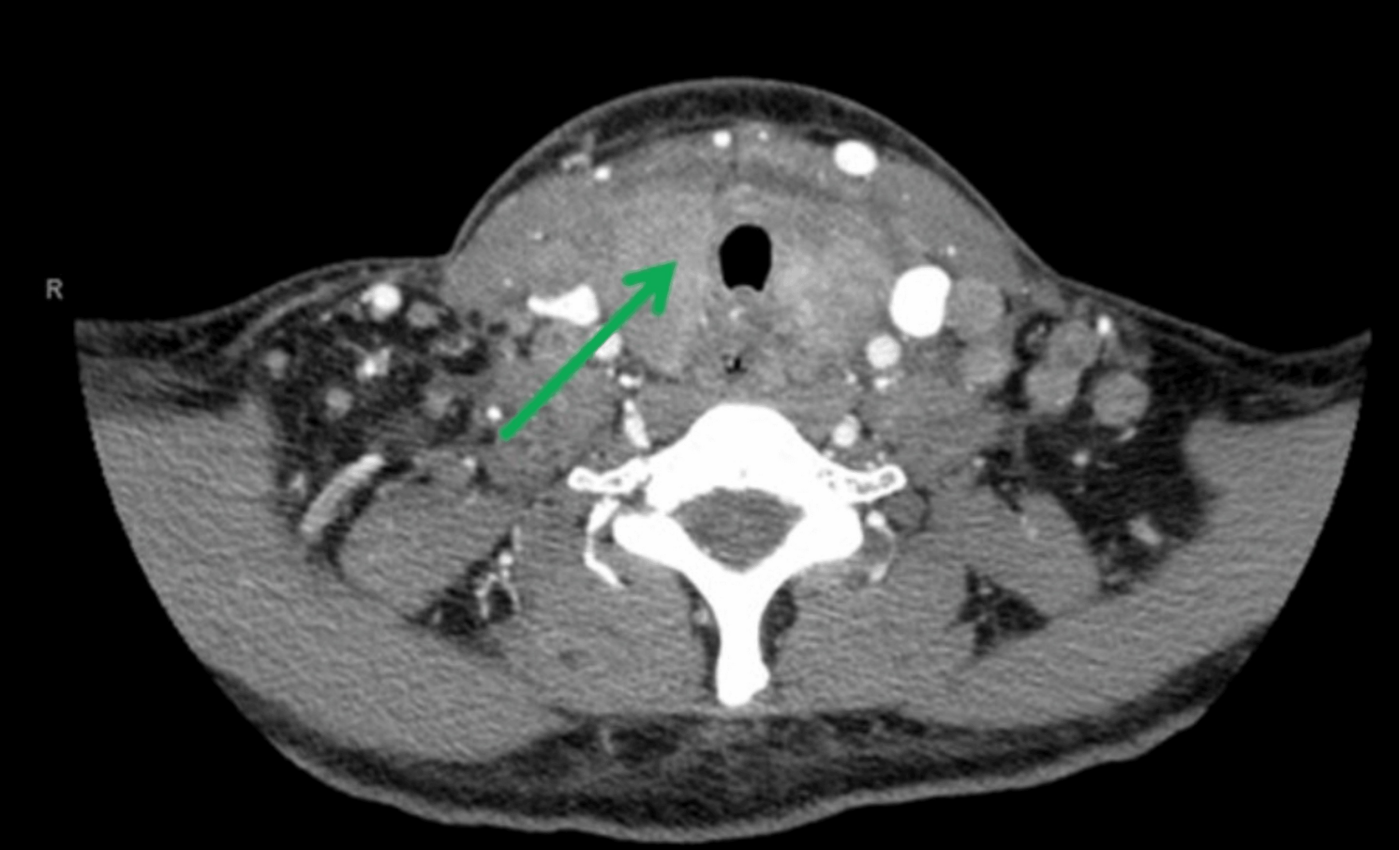
CT neck with contrast demonstrating indistinct thyroid gland (green arrow).

**Figure 3:**
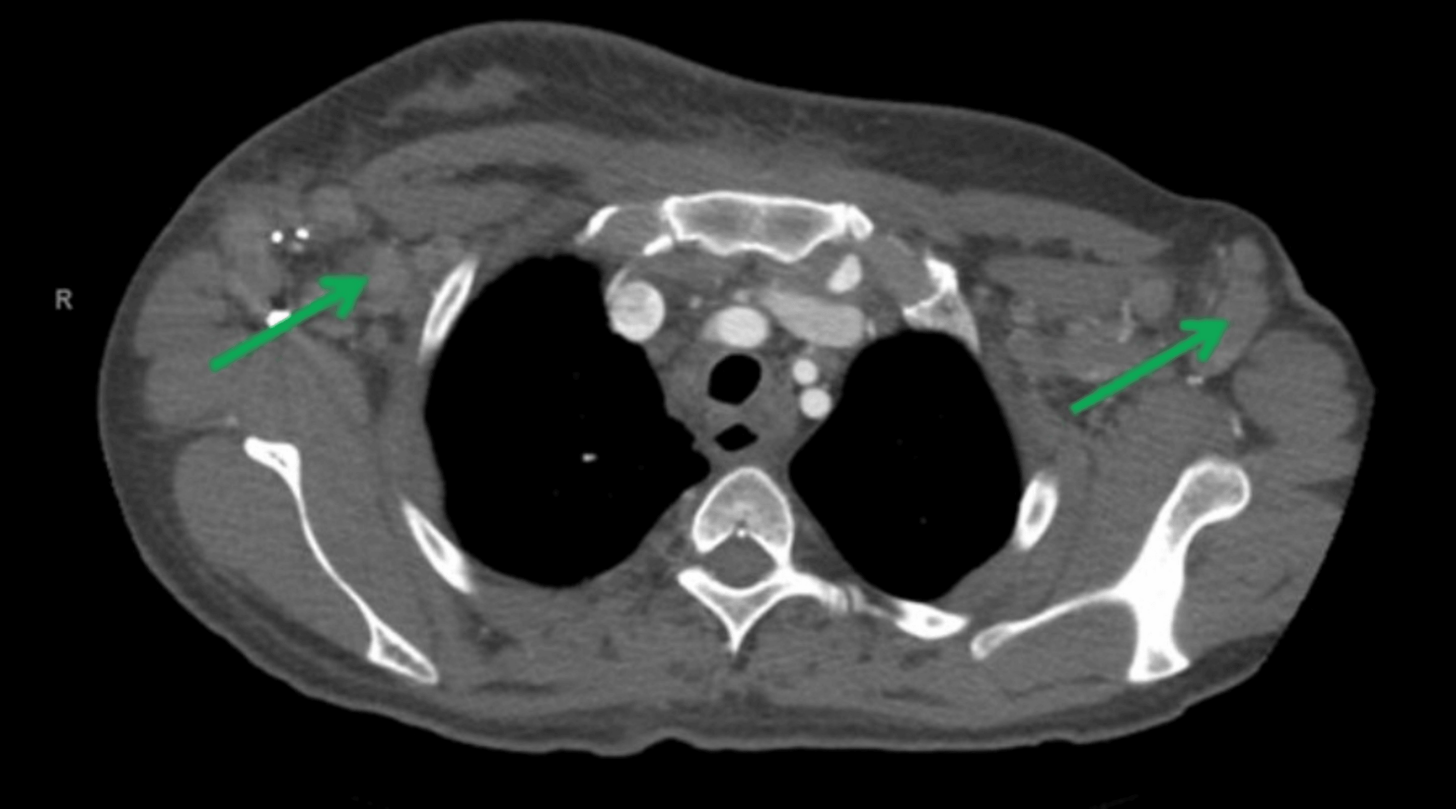
CT chest with contrast demonstrating axillary adenopathy (green arrows).

**Figure 4:**
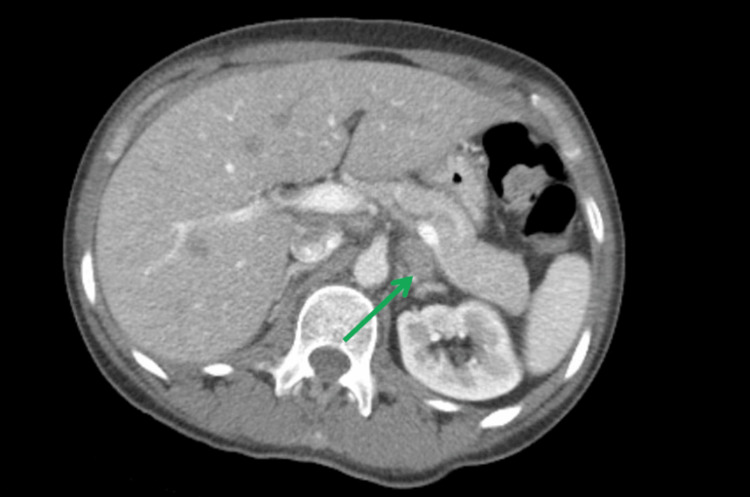
CT abdomen/pelvis with contrast demonstrating enlarged lymph nodes (green arrow) in the upper abdomen.

Following this, the patient was admitted for an extensive workup. Computed tomography (CT) neck with contrast showed diffuse bilateral level I-IV cervical lymphadenopathy, right greater than left, with no distinct masses (Figure 1). The thyroid gland was indistinct and enlarged (Figure 2). CT chest with contrast revealed generalized intrathoracic and axillary/subpectoralis adenopathy (Figure 3). There was no underlying breast asymmetry or mass identified. CT abdomen/pelvis with contrast (Figure 4) revealed intra-abdominal and retroperitoneal adenopathy with lymphadenopathy predominantly in the upper abdomen, suggestive of metastatic lymphadenopathy. 

**Figure 5:**
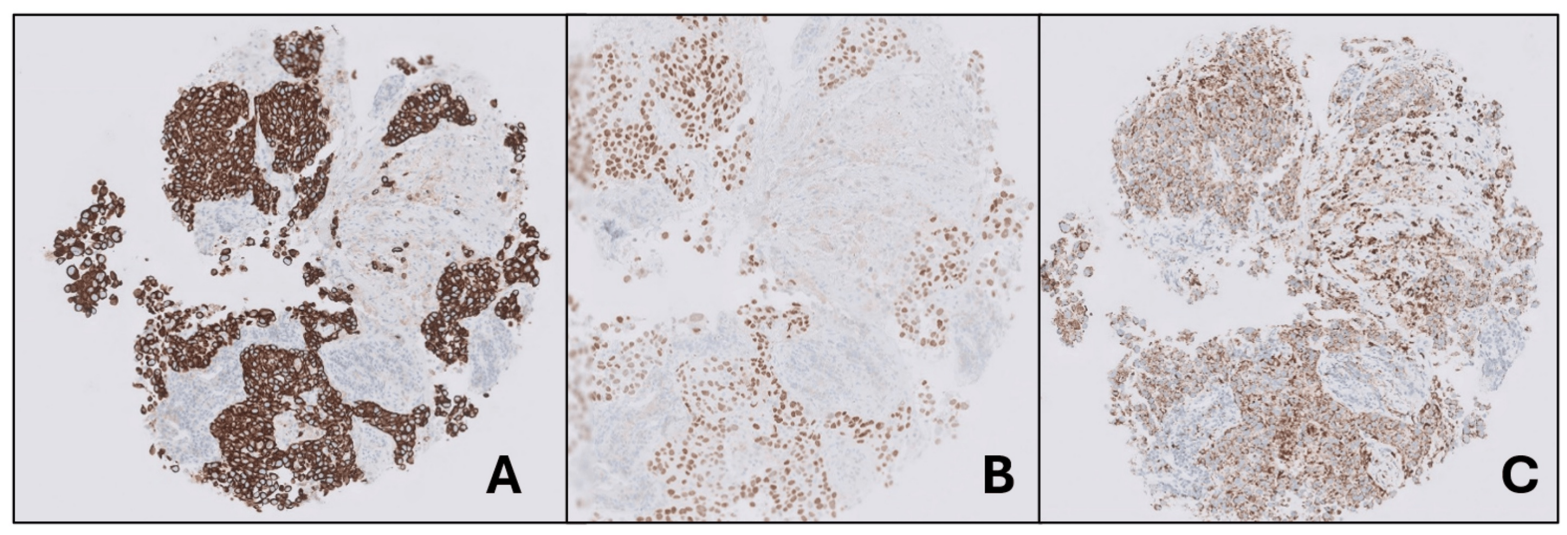
(A) CK7 stain, right supraclavicular lymph node biopsy. (B) TTF-1 stain, right supraclavicular lymph node biopsy. (C) Napsin-A stain, right supraclavicular lymph node biopsy. TTF-1: thyroid transcription factor-1

Ultrasound image-guided fine needle aspiration and core biopsy of the right supraclavicular lymph node were performed, and diagnosis of metastatic carcinoma with no confirmed single primary site was obtained. Core biopsies showed solid sheets of neoplastic cells with enlarged pleomorphic nuclei and prominent nucleoli with mitotic figures in a background of lymphocytes and desmoplastic stroma. Immunostains performed on the tumor cells within the cell block were positive for cytokeratin 7 (CK7) (Figure 5A), TTF-1 (Figure 5B), napsin-A (Figure 5C), and weak patchy for paired-box gene 8 (PAX8). The immunostain profile suggested that the main differential diagnosis for the primary origin of the tumor included lung adenocarcinoma versus thyroid carcinoma. Oncology and otolaryngology services were subsequently consulted, and the patient’s case was discussed at the multidisciplinary tumor board with recommendations for obtaining a positron emission tomography (PET) scan to further localize the primary tumor.

**Figure 6:**
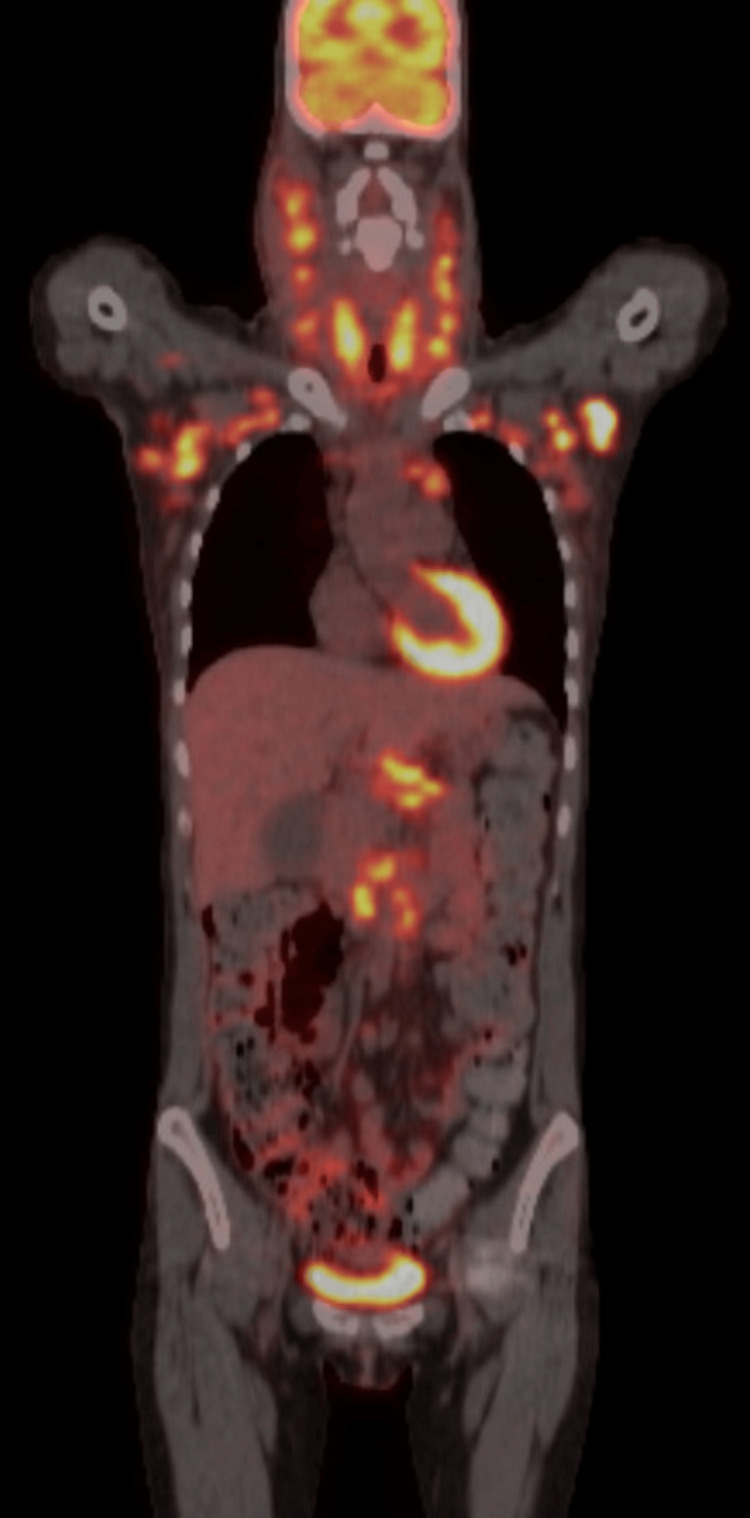
PET tumor imaging skull to thigh demonstrating diffuse, markedly hypermetabolic lymphadenopathy in the neck, chest, and retroperitoneum above the pelvis. PET: positron emission tomography

PET impression was concordant with the pathologic diagnosis of metastatic carcinoma with no primary malignancy identified (Figure 6). The thyroid was observed to be enlarged and diffusely hypermetabolic. There was no abnormal activity, suspicious nodule, or mass identified in the lungs. 

**Figure 7:**
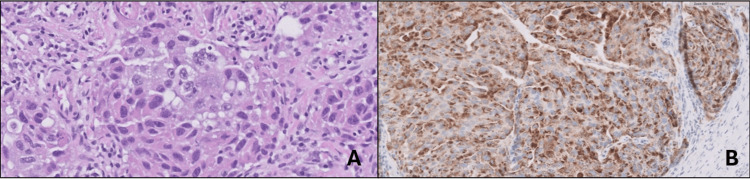
(A) High-power thyroid gland biopsy. (B) Napsin-A thyroid gland biopsy.

Shortly after discharge, the patient was re-admitted due to shortness of breath. The patient presented with worsening supraglottic edema and associated dyspnea eventually requiring tracheostomy. Otolaryngology performed a tracheostomy tube placement and a thyroid excisional biopsy due to concern for malignancy. Intraoperatively, the strap muscles were found to be firmly adhered to the underlying thyroid gland. Additionally, dissection of the thyroid isthmus revealed thickened tissue. Pathology of the thyroid gland samples revealed metastatic adenocarcinoma consistent with lung primary (Figure 7A). Immunostains showed positive for napsin A and weak, patchy positive for TTF-1 (Figure 7B). The patient was subsequently diagnosed with stage IV lung adenocarcinoma with metastasis to the thyroid gland. 

The patient was started on chemotherapy with carboplatin/pemetrexed and pembrolizumab with plans for further follow-up with medical oncology. During treatment with chemotherapy, the patient presented with headache, neutropenia, and anemia. CT head with and without contrast was performed in which no evidence of metastasis was observed. She subsequently followed up with Otolaryngology and was found to be breathing well. Flexible laryngoscopy revealed mild hydropic edema of the aryepiglottic folds, arytenoids, and post cricoid region. Decision was made to continue with the tracheostomy tube until treatment with chemotherapy was completed. The patient was subsequently lost to follow-up after discharge.

## Discussion

This case highlights the importance of a thorough diagnostic workup for primary localization for metastatic carcinomas. Diagnosis of a secondary neoplasm of the thyroid gland has critical implications for patient management and prognosis [6]. Management of secondary thyroid neoplasms compared to primary thyroid neoplasms differs significantly [6]. Studies have shown that nonrenal secondary thyroid neoplasms have worse outcomes with rapid dissemination and more commonly present with stage IV diseases [6,7]. For lung carcinoma, in particular, the average survival from diagnosis to death in patients with metastatic disease to the thyroid is two months [8]. Consequently, despite the rarity of lung adenocarcinoma metastasis to the thyroid, consideration of the thyroid during clinical diagnosis is crucial to determining appropriate treatment.

One of the key highlights of this case is timely diagnostic workup. Given the patient presentation at the time, highest on the differential was lung and thyroid cancer, with considerable effort made to determine the primary neoplasm. Multidisciplinary discussions initially recommended a PET scan to further localize the primary site. Then, if the diagnosis was unclear, FNAB of the thyroid gland could be considered. Ho et al. conducted a retrospective study in which 4,281 cases used PET to determine the initial stage in all malignant tumors [9,10]. They found that further tests such as ultrasound or FNAB of the thyroid were recommended if focal thyroid uptake was first detected by PET [10]. FNAB of the thyroid gland is a sensitive and accurate method for detection of secondary thyroid neoplasms [2]. FNAB is helpful in cases for confirming a suspicion of local recurrence or metastasis of a known primary cancer [11]. However, diagnosing metastatic lesions without an obvious primary site is challenging [11]. For this case, FNAB was considered a secondary option given that the thyroid was diffusely enlarged thus making it challenging to identify specific sites for biopsy. Given the patient’s acute onset of symptoms following PET and the need for tracheostomy, an excisional thyroid biopsy was performed at the time of tracheostomy and aided in subsequent diagnosis. Immunocytochemistry with various antibodies as a panel, including TTF-1, PAX8, CK7, and CK20, is helpful in the confirmation of a secondary thyroid neoplasm diagnosis as observed in this case [2]. However, TTF-1 is a tissue-specific transcription factor [12]. While it is one of the molecular markers for the diagnosis of lung tumors, its expression is not specific and can be seen in thyroid or other organ origins. Napsin A is a marker of primary lung adenocarcinoma with 96% specificity [13]. PAX8 is not detectable in primary lung adenocarcinoma but is expressed in more than 90% of thyroid carcinomas [13]. While there are a limited number of reports describing primary lung adenocarcinoma with metastasis to the thyroid, some reports have documented similar clinical features. Like our case, these cases also presented patients with a smoking history and lymphadenopathy of the neck, mediastinal, and supraclavicular regions [14-16]. CT imaging revealed diffuse lymphadenopathy, FNAB of suspicious lymph nodes revealed malignant nuclear features and pathology specimens resulted in similar tumor markers [14-16]. As expected for a typical pulmonary adenocarcinoma, immunohistochemistry for these cases was positive for TTF-1, napsin A, and CK7 and negative for PAX8 and CK20 [13-16]. One case that resulted in thyroidectomy also observed an enlarged and densely adherent thyroid gland to the overlying strap muscles [17]. In contrast, while imaging for our patient did not reveal any masses or nodules in the lung or thyroid, one case reported a patient in which CT chest revealed a nodule without apparent lymphadenopathy [7]. Furthermore, other cases presented patients with no smoking history and initial rapid enlargement of the thyroid [8,9,18]. Our patient was also in her third decade of life, whereas other cases, more consistent with the literature, were in their fifth to seventh decade of life. Additionally, other authors presented cases of thyroid metastases from pulmonary origins with squamous cell carcinoma, non-small cell carcinoma, and anaplastic small cell carcinoma histologic types [19]. Few cases were adenocarcinomas, as they typically induce metastases to the liver, adrenal, bone, and brain [19]. In addition to chemotherapy treatment, management of metastases to the thyroid gland depends on the dissemination and advanced stage of the primary tumor. Surgical management is considered on a case-by-case basis. Ishikawa et al. suggested that thyroidectomy was indicated for patients whose metastasis was limited to the thyroid to prevent further dissemination of the primary tumor [7]. It was also considered for patients who experienced dysphagia or dyspnea to improve quality of life [7]. Moreover, the study suggested that thyroidectomy as treatment for metastasis to the thyroid gland does not necessarily improve survival time [7]. Therefore, accurate prompt primary localization of metastatic carcinomas to guide treatment plans is key.

## Conclusions

Metastasis to the thyroid gland due to primary lung adenocarcinoma is a rare occurrence. Diagnostic steps are guided by imaging, including CT, PET, and FNAB, and immunocytochemistry findings. Accurate and timely diagnosis of secondary thyroid neoplasm significantly impacts patient management and outcome. Consequently, it is critical to keep the thyroid gland in mind as a site of metastasis when evaluating diffusely metastatic carcinoma. 
